# The perception of risk in contracting and spreading COVID-19 amongst individuals, households and vulnerable groups in England: a longitudinal qualitative study

**DOI:** 10.1186/s12889-023-15439-8

**Published:** 2023-04-05

**Authors:** Kerry Hanna, Pam Clarke, Kerry Woolfall, Shaima Hassan, Katharine Abba, Taghreed El Hajj, Elizabeth Deja, Saiqa Ahmed, Neil Joseph, Adele Ring, Gerry Allen, Paula Byrne, Mark Gabbay

**Affiliations:** 1 Health Sciences, University of Liverpool, Thompson Yates Building, Brownlow Hill, Liverpool, L69 3GB UK; 2 Department of Primary Care and Mental Health, University of Liverpool, Liverpool, UK; 3 Department of Public Health, Policy and Systems, University of Liverpool, Whelan Building, Liverpool, UK; 4 NIHR Applied Research Collaboration NWC, Liverpool, UK; 5 Liverpool School of Tropical Medicine, Liverpool, UK

**Keywords:** COVID-19 pandemic, Inequalities, Public involvement, Risk, Pandemic restrictions

## Abstract

**Background:**

Social distancing restrictions to manage the COVID-19 pandemic were put in place from March 2020 in the United Kingdom (UK), with those classed as “highly clinically vulnerable” advised to shield entirely and remain at home. However, personal risk perception has been shown to comprise of various elements beyond those outlined in the national pandemic guidance. It is unclear whether those deemed COVID-19 vulnerable identified as high-risk to COVID-19 and thus complied with the relevant advice. The aim of this research is to explore the perception of risk in catching and spreading COVID-19, amongst individuals from individual households, and vulnerable groups in a region of the UK.

**Methods:**

Two individual, semi-structured interviews were conducted, four-weeks apart, with adults living in households in the Liverpool City Region. At the follow-up interview, participants were given the option of using photo-elicitation to guide the discussion. Reflexive thematic analysis was employed to conceptualise themes. The qualitative analysis was underpinned with symbolic interactionism.

**Results:**

Twenty-seven participants (13:14 males:females, and 20 with a vulnerable risk factor to COVID-19) completed a baseline interview, and 15 of these completed a follow-up interview four-weeks later. Following thematic analysis, two overarching themes were conceptualised, with subthemes discussed: theme 1) Confusion and trust in the risk prevention guidance; and theme 2) Navigating risk: compliance and non-compliance with public health guidance.

**Conclusion:**

Participants developed their own understanding of COVID-19 risk perception through personal experience and comparison with others around them, irrespective of vulnerability status. COVID-19 guidance was not complied with as intended by the government, and at times even rejected due to lack of trust. The format in which future pandemic guidance is conveyed must be carefully considered, and take into account individuals’ experiences that may lead to non-compliance. The findings from our study can inform future public health policy and interventions for COVID-19 and future pandemics.

**Supplementary Information:**

The online version contains supplementary material available at 10.1186/s12889-023-15439-8.

## Background

From 31^st^ December 2019, severe public health restrictions were announced around the world to tackle the spread of the COVID-19 pandemic [1]. In the UK, citizens were classed as vulnerable to the virus if they were > 70 years old or had pre-existing health conditions that affected their immunity [2]. National social distancing restrictions were put in place from March 2020, with those classed as “highly clinically vulnerable” advised to shield entirely and remain at home [2, 3]. Risk perceptions are *“beliefs about potential harm or the possibility of a loss. It is a subjective judgment that people make about the characteristics and severity of a risk”* [4]. Risk perception refers to people’s intuitive evaluations of hazards that they might be exposed to [5]. However, the literature suggests that risk perception consists of various elements, including individual, societal, cultural, and contextual factors [6]. It is therefore, unclear whether the members of the public who were deemed COVID-vulnerable identified as high-risk to COVID-19 and thus, adhered to the relevant advice.

There is evidence of variation in how different groups of people consider themselves at risk from COVID-19. People from ethnic minority backgrounds were disproportionally affected by COVID-19 [7]. A qualitative study exploring COVID-19 risk perception in Muslim community members reported that risk perception related to perceptions of exposure to the virus, through factors such as employment [8]. Surveys in Germany and the USA found that women, older people [9], and individuals with a higher educational level [10] reported themselves to be at higher risk from the virus, despite the evidence being that those from poorer, less educated backgrounds were likely to be at higher risk than their better resourced peers, and consequently undertaking greater engagement in protective behaviours. However, these studies failed to consider possible cultural factors, and the brief, self-report nature of surveys limits in-depth exploration as to why these findings occurred [10]. Nevertheless, these findings raise the question as to whether individual groups interpret their own health risk differently to others, and how this may affect their compliance with national COVID-19 guidance.

The results of national pandemic-related restrictions and isolation measures have been detrimental to many individuals, and these experiences may further influence their perception of risk. Vulnerable groups including those with caring responsibilities, have been particularly impacted by the pandemic, and have experienced significant impacts to their mental and physical health during the pandemic, due to increased financial insecurity and demands of home schooling whilst working from home [11]. In a further survey following lockdown restrictions in the UK, it was reported that disabled people were more likely to experience reduced working hours and higher levels of financial stress [12], although this research was limited by a poor survey response rate. In addition, Disadvantaged people in the UK, living in areas of multiple deprivation, experience exacerbated issues with unstable working conditions, greater rates of overcrowded accommodation, poor housing conditions, limited access to personal outdoor spaces, and reliance on public transport, meaning they are less likely to comply with national social distancing directives [13, 14]. The impact of these vulnerability factors can reach beyond the risk of COVID-19, as research has found greater rates of stress, anxiety, depression and a lack of sleep, as a result [15]. Thus, we may deduce that “vulnerable” status during the pandemic expanded beyond the COVID-19 health-risks described in the government guidance. High-quality, in-depth research, including a range of participants, is warranted to obtain a better understanding of individuals’ perceptions of risk and subsequent risk-prevention behaviours during pandemics.

The current evidence base is saturated with surveys conducted quickly during the height of the pandemic which, therefore, lack insight into people’s views and motivations to comply with COVID-19 guidance. Consequently, an inclusive, in-depth analysis on individuals’ views and perceptions around the risk of catching and spreading COVID-19, is needed. Thus, the aim of this longitudinal, qualitative research is to explore the perception of risk in catching and spreading COVID-19, amongst people from individual households, and vulnerable groups in the UK. Our main research question is: *How did individuals and vulnerable groups perceive risk of catching and spreading COVID-19 during the pandemic?* We believe that the findings can inform future public health policy and interventions for COVID-19 and future pandemics.

## Methods

Longitudinal qualitative research was chosen for this study, as it has been shown to support understanding of experiences across time and to identify facilitators and inhibitors of health and illness behaviours and transitions [16]. Specifically, we conducted two sets of semi-structured interviews, four-weeks apart, with adults (≥ 18 years) living in households in the Liverpool City Region: an area of relative economic and social deprivation in the UK. At the follow-up interview, participants were given the option of using ‘photo-elicitation’ to guide the discussion [17].

### Background context

**Fig. 1:**
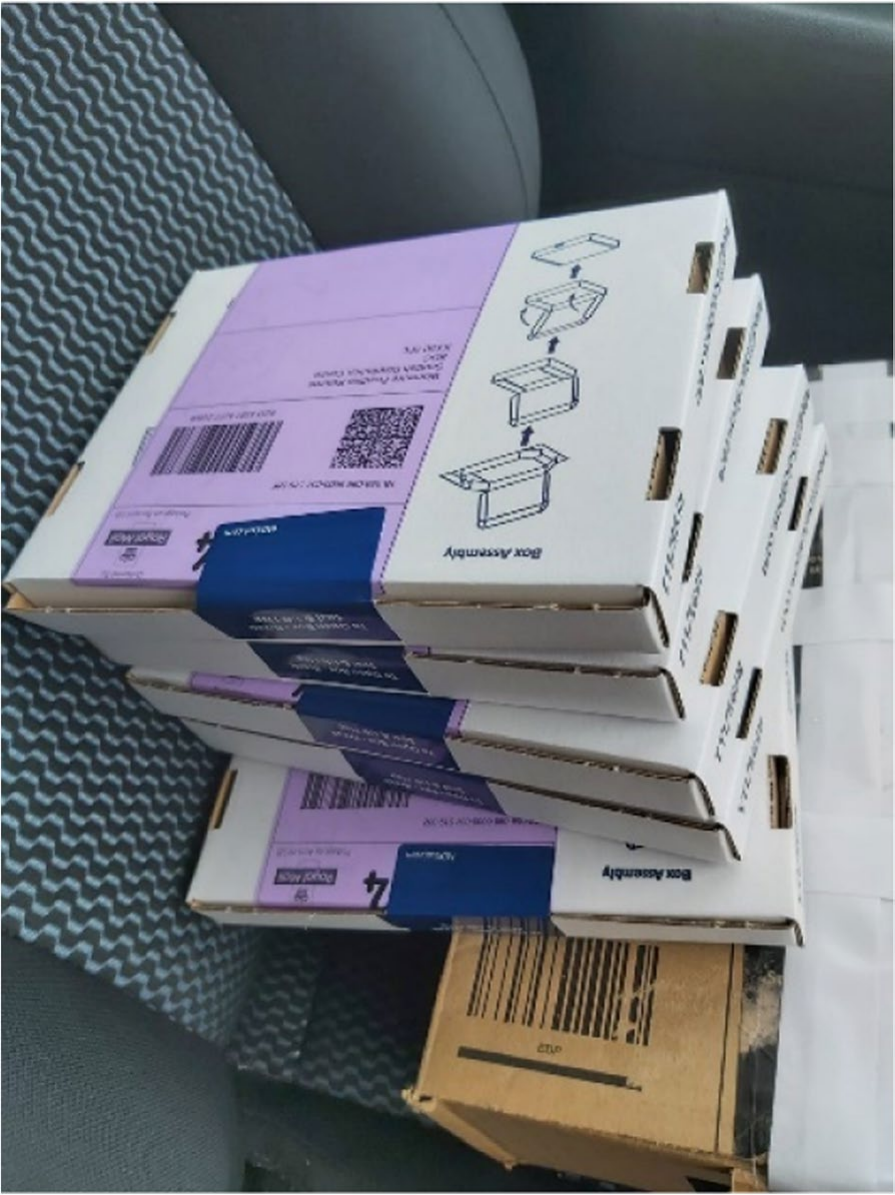
Image taken by participant showing COVID-19 lateral flow tests

Interviews were conducted between July and November 2020. Before and during this period there were various changes to COVID-19 legal restrictions on movement and social contact as infection levels peaked in the spring, fell during the summer and rose again in the autumn. During this time there were also changes to COVID testing availability and rules, and rules regarding self-isolation, international travel, and the wearing of face coverings (Fig. 1).

Data collection began during a period of falling infections and relaxation of restrictions and ended during a period of rapidly rising infections and shortly before a second national lockdown began (in England). See Supplementary Material 1 for context of restrictions before and during the study.

### Sampling and recruitment

Participants were purposively sampled from respondents to a longitudinal survey “COVID-Liverpool Household Survey: The Psychological and Social Impact of COVID-19 (COVID-Liv A)" [18]. These respondents indicated their willingness to be contacted by researchers working on other COVID-19 studies. The sample for COVID-Liv A was drawn initially from the CLAHRC NWC Household Health Survey, which surveyed adults over the age of 18 living in households in the study area. Recruitment for COVID-Liv A was later expanded to include individuals targeted through social media campaigns.

The COVID-Liv A survey was also used to recruit participants in a viral surveillance study (COVID-Liv) involving weekly COVID testing. All participants in the current study had therefore also participated in an online questionnaire survey, and some had also taken part in a weekly COVID testing study. Demographic data collected during the COVID-Liv A survey was used to identify potential participants for this sub-study in the following categories of interest: people living alone; people living with young children; people with caring responsibilities; people who identify themselves as vulnerable to COVID-19 due to a health condition; people aged over 70; and people not falling into any of the previous categories. The chosen categories were derived from the UK government and health service’s definitions of health inequalities and those deemed high risk from COVID-19 [2, 19, 20]. We used purposive sampling with the aim of recruiting a balance of males and females representing the full range of categories of interest. Of note, subsequent unpublished sub-studies of this overarching work, were conducted to explore risk perception by communities and organisations.

Potential participants were initially contacted by email which included an invitation letter, copies of the study information sheet and consent form, and contact details for members of the research team. This was followed by a telephone call around a week later, at which point the researcher answered any questions the person might have and, if they were willing to take part, arranged a time and means (telephone, or online/Zoom) for the interview that was convenient for them, and during a time where they could speak privately without other people present.

### Data collection

The COREQ quality checklist for qualitative research, has been used to guide the reporting of this research [21]. Baseline interviews were conducted between July and October 2020). An optional follow-up interview, with or without photo elicitation, was offered to all participants, approximately four-weeks post-baseline. Follow-up interviews took place between September and November 2020, and aimed to explore changes to risk perception and individual experience over time. The researchers made additional field notes during the interviews. The purpose of the research was explained, and written and verbal consent were taken prior to the interviews commencing. A pre-designed topic guide, co-developed with our public advisors (see Appendix 1), allowed the research team who conducted the interviews (KH (PhD), PC (MPhil), KA (PhD), SH (PhD), THE (PhD) & MG (MD FRCGP)) to follow a semi-structured format. All interviewers were experienced in interviewing for qualitative research. Interviews were offered via phone or Zoom according to the preference of the participant, and were audio recorded. Audio recordings were transcribed verbatim by an experienced transcriber at the University. Transcripts were not returned to participants for accuracy as no recording errors were identified. Research team discussions were used to support analysis and conceptualisation of themes. Due to the constantly changing circumstances of the pandemic, new data was regularly being collected at each new interview. Thus, the research team deemed it appropriate to stop recruitment after the UK had entered a second lockdown, and a spread of participants from various backgrounds and vulnerable groups had been recruited to ensure a range of experiences were captured. Data saturation was also noted when the UK entered a second lockdown, and participant accounts showed similarities to those after entering the first lockdown. Interviews lasted on average 38.1(± 13.3) minutes at baseline, and 35.5(± 15.8) minutes at follow-up.

### Photo elicitation

Participants who continued to follow-up were given the options of using ‘photo-elicitation’ to guide the discussion, or take part in another interview without photographs [22]. The former option was introduced for participants, as it has been reported that some people find it easier to express themselves through their chosen images, allowing them time to reflect on their experiences prior to the interview [22]. Furthermore, to avoid exclusion of participants who would not feel comfortable taking and sharing photographs, or who did not own a suitable device (as is possible amongst some vulnerable groups in the UK [22, 23]), this method was not a requirement for a follow-up interview. Consented participants were asked to use their own camera or mobile phone to take photographs that were meaningful to them and represented their thoughts about the pandemic. Participants took photographs during the four weeks prior to their follow-up interview and emailed copies of the photographs they had selected to talk about before the interview, which were then used to capture the narratives created during the discussion. Specific guidance was provided, including the safety and ethics of taking photographs, and the researcher also provided instruction and support by telephone. For continuity, baseline and follow-up interviews were conducted by the same researcher.

### Data analysis and epistemology

Our approach to qualitative analysis was iterative, informed by symbolic interactionism [24]. This sociological theory underpins the exploration of behaviour and social roles, enabling an understanding of how people interpret and react to their environment which, in the context of this study, relates to their environment under COVID-19 restrictions. Thematic analysis (TA) is a method of identifying, analysing and reporting patterns within data [25]. TA was chosen for this research as it offers a flexible and in-depth way of exploring the research question, and allows us to consider not only the perspectives of the individuals, but the meanings behind their choices and the impact of the wider social environment on these meanings [25]. A reflexive TA strategy was followed, based upon the six-step model outlined in Clarke and Braun [26]. Once familiar with the data, initial analysis [27] was employed by the research analysis team to generate preliminary codes, alongside consideration of symbolic interactionism. The research team discussed initial codes, and from this work, devised an overarching coding frame, using the NVivo 12 software [28], to further support organisation of the emerging codes. However, final thematic development occurred after all transcripts had been coded. The frame ensured that coding remained focused on the research aim and theoretical underpinning, and was agreed by the wider team, consisting of academics, clinicians and public advisors. On completion of the coding, themes and subthemes were developed through research team discussions with the wider team and public advisors.

### Reflexivity

Reflexivity ensures quality and rigour in the research, and the researchers’ position is important when considering similarities or differences among the participants [29, 30]. The authors have a varied range of professional backgrounds (in academic teaching and/or research (KH, PC, MG, KA, KW, SH, TEH, ED, AR, PB), medicine (MG), sociology and social science (KW, ED, TEH, KH, SH, PB), allied health care (KH), primary care (MG) and public advisors/involvement (NJ, SA, GA)). KH, PC, MG, KA, KW, SH, TEH, ED and AR are experienced qualitative researchers, and KH, KW, PC, ED and MG coded the transcripts. Ten authors are female and three are male. All authors involved in this study have a research interest in addressing socio-economic health inequalities. The non-academic authors were given training in qualitative research, and were closely supported in analysing the data for this study. Thus, all authors have contributed their differing perspectives in research discussions to agree the overall findings, and all authors contributed to team-based reflection in order to strengthen the development of the study documents, the data analysis and interpretation, and approval of the manuscript.

### Public involvement (PI)

We have used elements of the GRIPP2 checklist to guide appropriate inclusion of PI in this project [31]. The role of PI in the study was to support targeted recruitment within the overarching study, and strengthen the data analysis, as our public advisors have first-hand experience of living in the communities, and experiencing the impacts, outlined in this study more so than the academics. Three public advisors were involved in all aspects of this sub-study, which consisted of co-production of documents (including the topic guides), transcript coding, group discussion about interpretation and thematic categorisation (whereby themes were conceptualised), and co-authorship of outputs. Formal training was provided, and public advisors were closely supported in undertaking qualitative analysis.

### Thematic analysis

Our main findings consider individuals’ understanding of the public health guidance, and their subsequent response in navigating risk and compliance/non-compliance with the public health guidance during the pandemic. Following TA, two overarching themes were conceptualised, with subthemes discussed within these: theme 1) Confusion and trust in the risk prevention guidance, and theme 2) Navigating risk: compliance and non-compliance with public health guidance. Table 1 shows the coding tree and subthemes following analysis.

**Table 1:** Coding tree of themes and sub-themes following thematic analysis

Themes	Sub-themes
1. Confusion and trust in the risk prevention guidance	• *Individuals’ knowledge and (mis)understanding of COVID-19* • *Rapidly changing guidance and trust in the government* • *Media influence on risk perception, and the rise in fake news*
2. Navigating risk: compliance, incompliance and non-compliance with public health guidance	• *Low perception of individual risk, and the advantages of complying* • *The perceived threat to others, and a responsibility to protect* • *Blaming and shaming others*

Of note, the government guidance on COVID-19 restrictions in England moved between guidance and regulations during the time of the study, and so we appreciate that at times, other terms such as “adherence”, may be deemed a more suitable term for breaching guidance rather than law. However, for consistency, we only use the term “compliance”, as compliance was the government expectation, backed by law enforcement and fines for many measures, including mask wearing, travel restrictions, household bubbles, and parties.

## Results

We recruited 27 participants for baseline interviews, 15 (55.5%) of whom completed a follow-up interview within one month. Two of these participants further consented to using photo elicitation to support their discussion during their follow-up interview. We considered that data saturation was achieved by interview 27, ceasing further recruitment. Table 2 shows the demographics of included participants. The Index of Multiple Deprivation (IMD) deciles show that many participants resided in relatively deprived areas [32], with the North West of England already representing greater levels of area derivation in relation to the South of England [33].

**Table 2:** Demographic characteristics of the recruited sample

N (%)	Participants at Baseline (*n* = 27)	Participants at follow-up (*n* = 15)
Gender		
Female	13 (48.1%)	6 (40.0%)
Male	14 (51.9%)	9 (60.0%)
Ethnicity		
White British	25 (92.6%)	14 (93.3%)
White Other	2 (7.4%)	1 (6.7%)
Ethnic Minority	0	0
IMD decile		
1 (most deprived)	6 (22.2%)	3 (20.0%)
2	4 (14.8%)	2 (13.3%)
3	2 (7.4%)	1 (6.7%)
4	2 (7.4%)	1 (6.7%)
5	0	0
6	4 (14.8%)	1 (6.7%)
7	1 (3.7%)	1 (6.7%)
8	4 (14.8%)	2 (13.3%)
9	0	0
10 (least deprived)	0	0
Data missing	4 (14.8%)	4 (26.7%)
Age	59.0 (± 13.1) [27–83]	62.2 (± 14.2) [27–83]
**COVID-19 vulnerability risk factors**		
Lives with young children	5 (18.5%)	0
Has caring responsibilities	4 (14.8%)	3 (20%)
Has COVID-19 health risk^1^	6 (22.2%)	1 (6.7%)
Is > 70 years old	8 (30.0%)	6 (40%)
Has none of the above vulnerability risk factors	7 (26.0%)	6 (40%)

^1^COVID-19 health risk = obesity, cardio/pulmonary disorders, immunosuppression medications etc. [2]

**Fig. 2:**
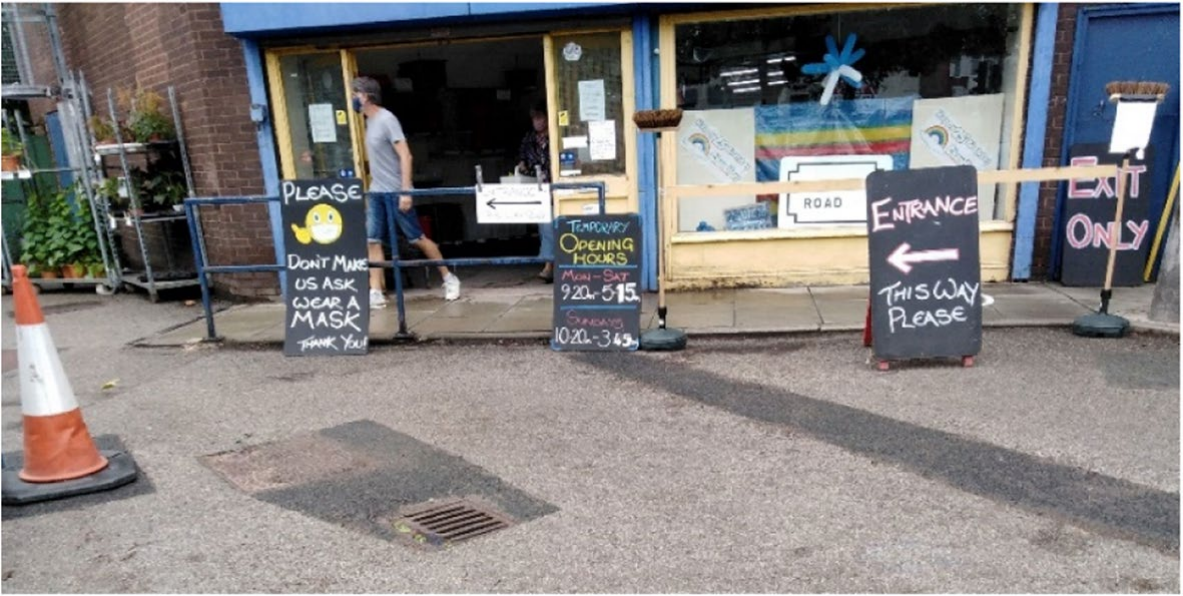
Image taken by participant showing sign outside public shop

**Fig. 3:**
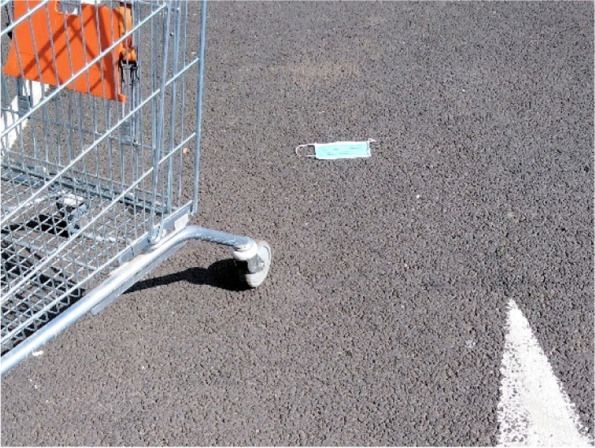
Image taken by participant showing face mask in supermarket carpark

**Fig. 4:**
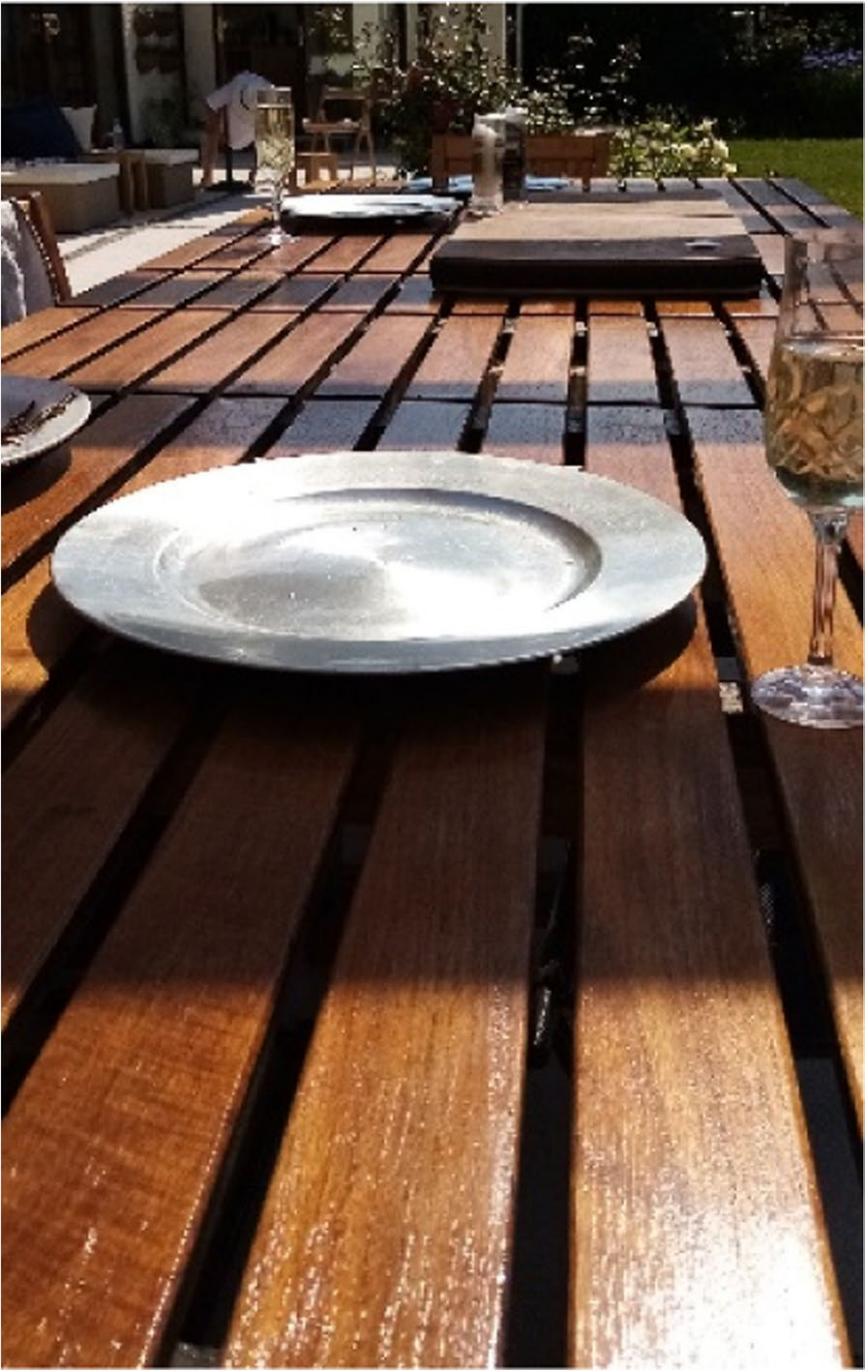
Image taken by participant showing outdoor dining

**Fig. 5:**
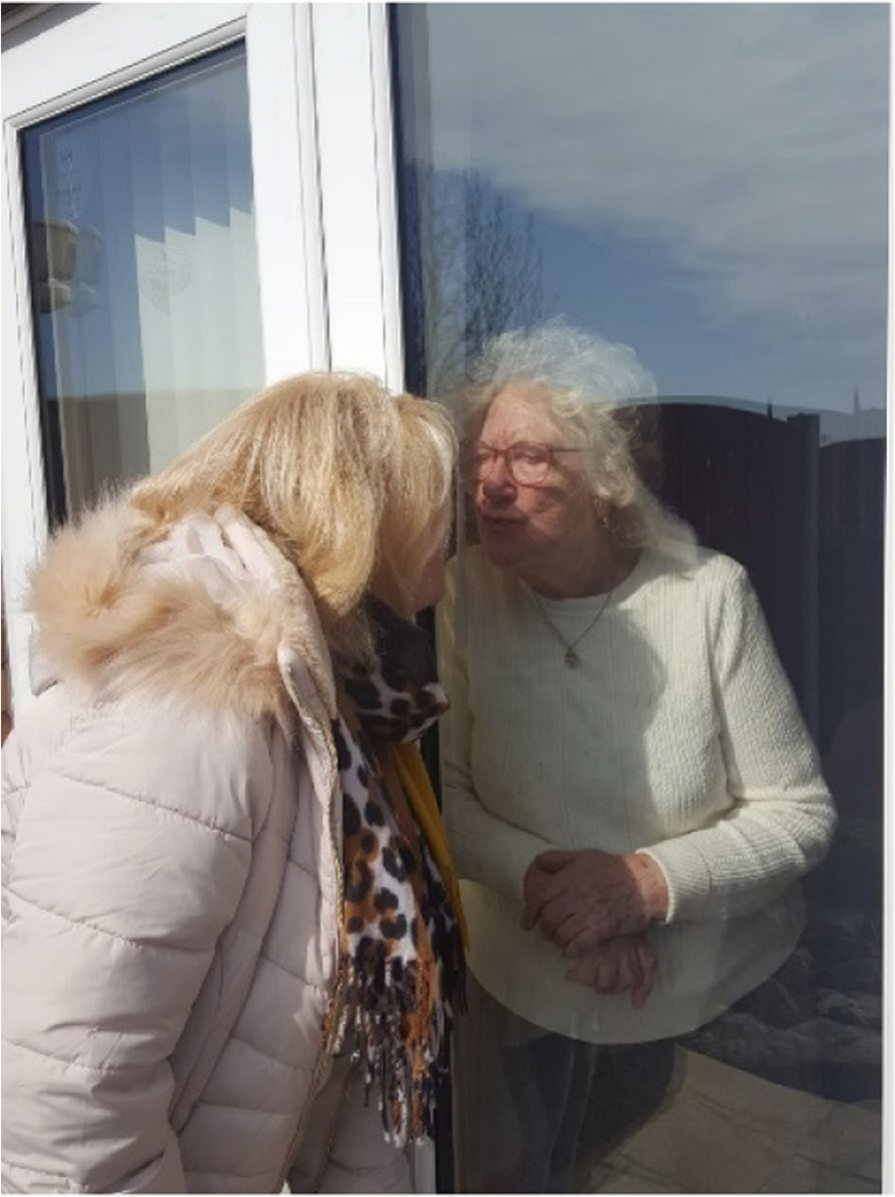
Image taken by participant showing visiting restriction during the pandemic

**Fig. 6:**
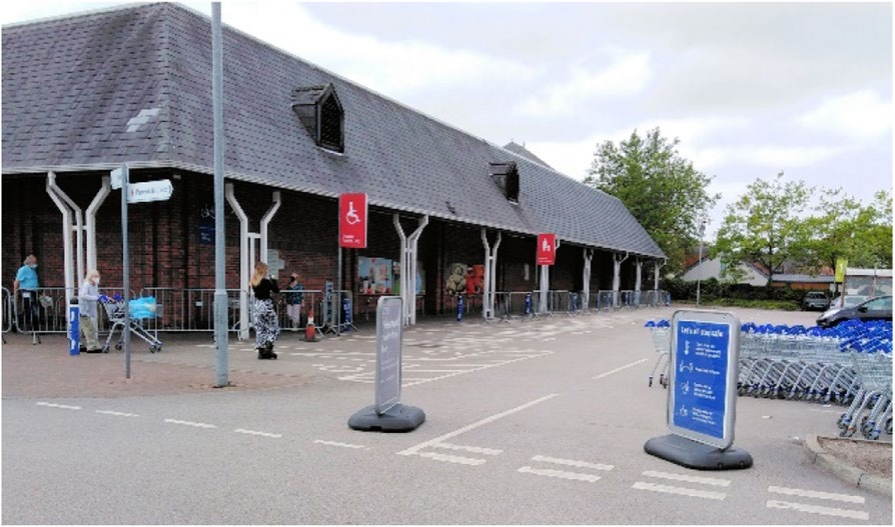
Image taken by participant showing supermarket queue

Through thematic analysis of the data we developed two themes and six sub-themes, as shown in the coding tree in Table 1. Where consenting participants used photo elicitation to support their interview discussion, these images have been included in the relevant subsections below (see Figs. 1, 2, 3, 4, 5, 6).

### Confusion and trust in the risk prevention guidance

This theme describes how participants understood the virus, and explored their sources of information. It was found that differences in understanding around the virus led to confusion, which in turn led to mistrust of information/sources. The media played a role in sharing (mis)information during this time, and participants acknowledged a rise in “fake news” circulating, that caused further confusion and the development of conspiracy theories. In addition, the government guidance during this time changed rapidly, both within the UK and globally, and further incidents occurred where government officials were caught breaching the guidance, which led to further mistrust in the way the country was being governed.

#### Individuals’ knowledge and (mis)understanding of COVID-19

At baseline interview, we asked participants to describe their personal understanding of the virus, which often led to accounts of how the virus spreads from one person to another, as well as their understanding of the public health guidance. It was apparent that many had differing views and understandings. The range of views described included, that the virus originated from China due to food consumption; that the risk of the virus diminishes outdoors; that the virus is airborne and can also be transferred through direct contact with contaminated objects; and that the virus takes between five seconds and 15 min to transmit.

It seems to be a deadly…strain of the flu…it’s airborne as far as I am aware, also through direct contact. So, if somebody has the virus and picks up a cup and then [I] pick up that cup after, there’s a chance [I would catch it]… 2. **P3_Baseline**

The way the coronavirus is transmitted, [our household] wanted to be away from people; particularly, we were apprehensive about joggers…they’re breathing heavily and…expelling more spores from the mouth… **P4_Follow-up**

Just from five seconds you can catch it…pretty much a five-second conversation at my front door I could catch it…if somebody’s got it and they knock at the door with a parcel… **P7_Baseline**

Factors that appeared to influence participants’ views of virus risk and transmission included information sources (such as word of mouth, television or government briefings), participants’ educational and/or occupational backgrounds, and the perceived complexity of the information given to them by the UK government. It was apparent that many participants sought information on the virus due to misunderstandings, or mistrust in the sources providing it.

I try to be a little bit wary of being too in my little bubble but…I’m fairly academic, I’ve worked to Masters level so I do understand a little bit about sources [laughs] and the ones which I check out seem to give you links to things like World Health Organization news and…medical views that aren’t the government’s spokes people who seem to have towed the political line a lot of the time… **P11_Baseline**

Many discussed conducting extensive online searches, and/or were actively involved in other research projects as participants. These participants stated that their reasons for being involved in research were to both better their own understanding of the virus, and to support the growing body of evidence aimed at tackling the spread of the virus. Thus, their views on the virus combined learned scientific knowledge, alongside their own personal views and experiences.

I have done my own research and I know that T-cells don’t show up…so even if you haven’t got the antibody it doesn’t mean that you haven’t had [COVID-19]…At the moment we don’t know whether…you can catch it again. But my own experience was my family and particularly my young grandson and the whole of his school had a cough for quite some time and I can distinctly remember him coughing right in my mouth…and I got ill after that… **P6_Follow-up**

To be honest, that [research study] gave me a sense of security that I know that I’m being tested on a regular basis and…I’m hoping by doing this that it helps people… **P3_Baseline**

#### Rapidly changing guidance and trust in the government

Central public health guidance discussed during interviews included social distancing, social isolation, social bubbles (the number of people one could interact with), and the wearing of face coverings/masks. Few participants felt they had a clear understanding of the information, with many reporting concerns in understanding what was being asked of them, and a general lack of trust in the government to respond appropriately. Participants observed that lack of clarity and loss of trust in the government guidance appeared to hinder the general public’s desire to comply.

Boris Johnson [UK Prime Minister] albeit too late…stood between two scientists…and he actually said something along the lines of, with a sombre expression on his face, that he’s very sorry to say that it’s inevitable that there will be a lot of people in your life, in your family, who will die before their time…That was a very hard-hitting message and since then it’s not been so hard hitting…. It’s been loosened so much so that the word on the street is it doesn’t matter, it [COVID-19]’s all gone, don’t worry about it… **P4_Baseline**

I think the [Dominic] Cummings thing…damaged a lot of people’s credibility but to be honest we’re stuck because where else do we go for our information if you can’t believe the government? **P6_Baseline**

We found that a lack of trust in the government resulted in breaching of guidance, even when later guidance was encouraging relaxed safety measures. Changing guidance, including the reduction of social distancing from two metres to one metre, left participants feeling unsafe and confused without clear reasoning. With feelings of uncertainty in the government guidance and leadership, participants reported making individual decisions, with many choosing to continue following the more stringent measures.

I have great difficulty in understanding the fact that they [the government] change the rules to suit the situation or suit the economic profile, so I maintain the two-meter distance because at the end of the day, it doesn’t matter which way the economy’s going, whether its driving ahead or whether we’re in recession, the transmission of this COVID19 disease will remain the same… **P4_Baseline**

When it was two metres…I felt quite safe and I went on the walks…and everyone was laughing about it sort of oh keep away from me…when it changed…from the two metre to the one metre [rule]…you’re not doing any social distancing because that conscious awareness had gone…I would have preferred it stay at two metres all the time to be honest… **P5_Baseline**

#### Media influence on risk perception, and the rise in fake news

Media sources, consisting of TV, radio, newspapers and social media, played a significant role in sharing important information about the COVID-19 virus and the government's changing public health measures. However, with a myriad of media information dominating everyday conversation, participants became increasingly confused over time as to what information was real and what was fake, resulting in many refusing to engage with media information at all and, as such, becoming unaware of any updates or changes to government guidance.

There is a vast amount of information out there, some of it to be honest is confusing…I got to the point…of not really watching the news because there were so many different stories coming out and…it was starting to get a bit depressing and nobody seemed to have a correct answer… **P3_Baseline**

I think there was lots of information…There was a tonne of information thrown at people and…it started to become white noise [“background noise”], that’s what it’s become to me, a lot of…white noise… **P15_Baseline**

Furthermore, some participants stated they had been reading COVID-19 conspiracy theories, due to the distrust and confusion in the official information provided. Others reported frustration in observing their friends and family indulging in theories, highlighting the influencing power of social media and fake news on the internet during this time. Ultimately, where participants did not trust that the virus was real, their perception of risk, and risk-prevention behaviours, appeared to diminish.

To be honest I felt that we were so ill informed I didn’t believe any of the information coming out, I thought that they [the government] should have been onto it [COVID-19] far sooner. I totally got into conspiracy theories. I was thinking this isn’t right, they have called it COVID-19 not COVID-20 so they know it’s been around…how could it possibly have taken this long to get the message through that this was a killer virus and it’s really affecting people very badly… **P6_Baseline**

I’ve got friends and I’ve kind of not fell out with them but…do you know these stupid people that believe in every…conspiracy, they think it’s 5G, it’s not coronavirus we’re getting killed by, [it’s] the 5G towers…It doesn’t matter how much you try to educate people they just, once they get that stuff in their head, that’s gospel truth… **P7_Baseline**

There's that much propaganda about it [COVID-19] on the telly and in the papers and on your phone, there's conspiracies now, everyone’s saying it’s all to do with pharmaceutical companies and you’ve just got to make your own decision… **P19_Baseline**

### Navigating risk: compliance, incompliance and non-compliance with public health guidance

This theme describes the participants’ response to the restrictive guidance in the UK, in terms of their decision to comply or not, due to their perception of risk. A finding from this section was the low perception of individual risk, compared to a higher perception of risk to others around them. Participants were influenced by the actions and views of people in their households, and communities, or as seen in the media, and made frequent comparison between their own actions, and those of others. Where it was noted that others were breaching government guidance, the participants expressed frustration and a desire for higher levels of policing (Fig. 2).

#### Low perception of individual risk, and the advantages of complying

Many participants did not believe themselves to be at risk of catching and/or spreading the virus, with a range of factors influencing this lack of individual risk perception. Where participants did not possess the published demographic/health indictors of those deemed most vulnerable to the virus (old age, obesity, ethnic minority status, cardiopulmonary conditions and compromised immunity), they described being less concerned and, therefore less compliant with guidance.

I’d hope that I wouldn’t [contract COVID-19] because I’m not obese or I’m not…an ethnic minority…and I’m under 70…I would hopefully be ok…So, I’m not in a high-risk group as such, although I do have a pre-existing medical conditional albeit mild… **P1_Baseline.**

Likewise, some reported feeling initially concerned about the virus, but perception of risk appeared to diminish over time where participants had not personally become ill with the virus, or failed to experience themselves/others to contract the virus. P2 below described contracting COVID-19 without symptoms, but at their follow-up interview, they reported feeling no worse than usual due to previous ongoing health issues, thus reducing individual risk perception.

I’ve got my own [pre-existing] health issues but generally speaking I’ve been pretty much ok really. I haven’t had any [new] symptoms of the COVID but already I do have good [and bad] days with me health, as it is normally…the conditions I’ve got…don’t put me in that position [at high risk]… **P2_Follow-up**

Furthermore, those who observed a lack of significant changes to their normal working day in comparison to the time before the pandemic often reported a low perception of individual risk that increased over time. Despite media reports highlighting global impacts to people’s lives, including the UK furlough scheme and requirements to work from home, many other staff were still required to carry on working as they did pre-pandemic. The lack of change (in contrast to the warning media reports) coupled with the lack of COVID-19 cases observed in their workplace, reduced risk perception and subsequent compliance with risk-prevention measures. This finding was particularly evident by the follow-up interview, and exemplified below by P3, whose perception of individual risk reduced as their employer’s guidance to comply with face coverings lessened over time.

For months we didn’t have face coverings…as far as my boss was aware, the government advice was that they weren’t compulsory so he didn’t get us them…[He] never gave us all direct guidelines on when we’re meant to wear them or not so because we’d gone through months not wearing them, even though we’ve got them most of us aren’t wearing them… **P3_Follow-up**

I’ve been going to work every day throughout, I use public transport, I have young children in school, so yes, I think there’s a reasonable risk that I could contract it or I may have already contracted it. I think I’m very low risk… **P15_Baseline**

No-one that I’ve actually worked with erm has had COVID… up to now I’ve not come across anyone with COVID…Apart from the restrictions that have been in place, has gone on mostly as normal… **P24_Follow-up**

Moreover, whilst some participants reported feeling “at risk” of the virus, their attitudes appeared to be influenced by the views and actions of their family, friends and colleagues. Even where participants admitted to disagreeing with others’ views, there was a strong desire to follow suit and avoid deviating from general public opinion. In these cases, participants reported breaching public health guidance to comply with social norms.

You try to think [about complying] but…you become relaxed and you follow other people…you mirror them and they get close to you and you don’t want to stand back and say oh get away from me you just sort of go into the flow…you don’t want to be rude to a person… **P5_Baseline**

For some, the public health measures were viewed primarily for navigating society under strict lockdown measures and so, compliance with guidance was described in terms of personal gain, rather than to reduce viral spread. For example, face coverings were worn by some participants in order to gain entrance to shops where this was required, but were not seen as a risk prevention measure at an individual level (Fig. 3).

I was in the supermarket today…I put a mask on to go in but as soon as I got in I took it off because only half the people in there had them on, even the staff never had them on so I don't know why they're telling people they’ve got to wear things in the shops when the staff aren’t wearing them… **P19_Baseline**

#### The perceived threat to others, and a responsibility to protect

In contrast to how risk was perceived at an individual level, participants reported that a greater risk was felt from the people around them. Participants described a sense of control for their own actions in preventing risk, but their inability to control the actions of those around them was a greater cause of concern. These participants further reported a sense of reassurance in the company of others who openly complied with the restrictions (Fig. 4).

The option to go out is still there for me and I take it, and…my wife…can go out if she wants…but I suspect a lot of what’s fuelling [my wife’s] side of it is the worry for her mother because…[COVID-19] will probably be a death sentence for her mother… **P3_Baseline**

I do feel safe…when I visit my friends, they’re all sort of careful, put the hand gel on, etc.*…* **P5_Baseline**

They [friends] had been isolating and I knew I’d be safe there … It was so hard not to hug … It was really rather sad… **P6_Follow-up**

As a result, compliance with public health guidance appeared to be greater in those with a caring responsibility, as participants feared they would transmit the virus to those more vulnerable. This included participants with a caring job role, or who voluntarily cared for another person in their community. Participants were mostly concerned for relatives and friends who were considered to have pre-existing health conditions classing them as “vulnerable”[2, 34], or who worked in areas of employment that involved exposure to others (Fig. 5).

[My son] was actually in a house with someone who got COVID because…the person he was living with was…a junior doctor… I was very worried… **P1_Baseline**

Certainly, we have more fears [for the family] than we have for our [household] because they’re younger, they work, they…expose themselves to a greater number of other people. So, there is a sort of a fear if you like at the back of my mind… **P4_Baseline**

We observed the social distancing like massively … We [could only look] through the window … We just can’t take the chance… **P6_Follow-up**

In addition to the aforementioned non-compliance, in which participants expressed a refusal to comply, additional reports of “incompliance” with social distancing directives emerged. In these cases, incompliance described participants’ perceived inability to comply despite awareness of the risks. It was identified that social isolation itself negatively impacted people, meaning participants felt they had no other choice but to breach guidance and visit their relative as a means of protecting their emotional wellbeing during the pandemic. Therefore, it was apparent that at times, the government directives were not deemed to be the safest or most practicable choice, and participants had to make their own decisions: whether to comply with public health guidance, or breach guidance and support their family’s wellbeing.

It wasn’t that long ago…she [daughter with mental health issues] said, please Mum, please can I have huggle and…I had to give her a hug, I had to, she was so low and…she washed her hands and did all of that [hygiene] and…so and it’s pretty much [remained] like that now **P6_Baseline**

#### Blaming and shaming others

Participants directly compared their own actions with those of people they considered to be less compliant, or more likely to spread the virus. Blaming language was often used to justify their actions, in instances where they admitted to breaching government guidance. Participants’ lack of compliance was minimised by their awareness of others complying to an even lesser degree. Often, participants compared demographics, such as ethnicity, age, health status, and lifestyle behaviours.

Young…people in unis are the ones that spread it far and wide because they have closer contact with each other because of their age group and…then they go and see friends and relatives and they give it to them and then the friends and relatives spread it a bit farther afield… **P2_Follow-up**

[The city] has a massive…financially viable Asian community: in other words, they can afford to buy tickets to and from… they have been bringing in the infection for three months before we locked down… **P18_Baseline**

Overtime, judgment between groups was widespread, affecting people on either side of the argument. Participants described feeling frustrated after observing others breaching guidance, and called for higher levels of policing to be enforced in order to control high rates on non-compliance. By follow-up, participants reported feelings of judgment by society, due to aspects of their demographic, where they chose not to comply with public health guidance (Fig. 6).

[Shop staff] have had no mask on…I’ve seen security fellas on the door with all the gear [PPE]…and people walking in with not a mask on and they haven’t stopped them…I’ll be honest, I had to go into [shop]…and I went straight for what I wanted and [immediately got] out…no, these were people [non-compliant shop staff] walking round putting stuff out and…they weren’t on the till where they have a screen… **P27_Baseline**

I think people would look at a person who’s older and think they obviously can’t wear a mask. But for someone younger, I don’t really want someone to start…saying to me, why aren’t you wearing a mask? So, I wear one, but I don’t feel very happy wearing one for very long… **P1_Follow-up**

Despite national guidance declaring that older age (> 70) made a person “high-risk” of severe impact from the virus, some older participants did not consider themselves to be more vulnerable due to their age, if they viewed themselves as healthy otherwise. These participants expressed frustration around younger people’s risk behaviours, declaring that the careless nature of younger people posed a greater threat to themselves. In addition, these older participants further blamed younger people for prolonging lockdown measures, due to persistently high rates of COVID-19 nationally.

No, I don’t think I’m more vulnerable…as I say, I’m 75, [but] I’m quite a young 75… **P16_Baseline**

I’d be perfectly happy knowing that I can socially distance and exercise all the logical and sensible precautions to not put myself in harm’s way but you see them [young people], everybody sees them. I only need to look at the TV…and see what I would describe as absolutely idiotic people [breaching government guidance]… **P4_Baseline**

I know they’ve got the facilities and everything else but not all [university] students are clean, some of them are dirty gits***,*** and they’re not bothered about spreading the disease; they think it’s fun…It isn’t fun for the people that get it…I think [students] should all be taken…[to] see people in intensive care with the lung ventilators…they should all be traipsed through there **P27_Baseline**

A similar attitude was identified among the younger participants, who agreed that they had a lower risk of severe impact from COVID-19 due to their age, and expressed concerns that they may be putting elders at risk. However, these participants worried that their elderly relatives were indeed, the deviant group, not doing enough to protect themselves from the virus.

Sometimes say like a neighbour’s kids will run up to me and just come for a hug or something and so, not to be mean to a kid I will just pick them up, which I know is…not within the letter of the guidelines but…you can kind of do the mental maths…[but] the elderly, it’s just not fair to them to put them in any risk… **P26_Baseline**

I went…to my parents’ [house] and… he [my Dad] said are you going to come in? I said no…because we are being told not to…and he said I find this very odd and surprising…I said…there’s rules in place for a reason…My parents complain about people round the corner…having parties… It’s OK to complain about other people not following rules than think about your own behaviour… **P22_Follow-up**

Overall, these accounts further highlighted the general loss of control that participants (of all ages) felt regarding the actions of others, in comparison to their own risk-prevention actions.

## Discussion

Our study was among the earliest to collect in-depth, qualitative data from participants in relatively deprived areas of the UK, who were declared both vulnerable and non-vulnerable to COVID-19. Published research reporting on risk perception and compliance have mainly relied on survey data and have failed to consider the perception of risk from those classed as most vulnerable to the virus [9, 10, 35]. Our study provides an in-depth exploration of risk perception, and how vulnerable and non-vulnerable individuals’ have subsequently complied with pandemic restrictions.

An international survey concluded that compliance with COVID-19 measures was significantly greater when there was a belief that following health precautions is effective in avoiding COVID-19 [36]. Our study echoes this finding; our participants described a high quantity of confusing information, which frequently changed between regions of the UK, with different guidance applied to different groups of people, such as those deemed vulnerable, and those working in jobs with higher rates of exposure. Despite this information showcasing the risk of the virus, and thus encouraging most to comply with restrictive measures, an “information overload” has been shown to deter people from watching, listening, or believing in media reports altogether [37]. Furthermore, previous qualitative research conducted in the UK has identified “alert fatigue” where individuals could not follow frequently changing rules, thus resulting in substantial non-compliance. Our study concurs with previous research, and further highlights that participants, unable to keep up with the high volume of changing information, chose what guidance they deemed fit to follow, based upon their own risk perception. In addition, Montiel et al. [38] highlighted the requirement for political leaders to adapt pandemic rhetoric to local societal conditions, whilst Moss and Sandbakken [39] suggested that leaders must also consider cultural differences, in order to secure public trust when persuading people to comply with measures. Therefore, to ensure compliance with future pandemic-related restrictions, governments must consider how messages are being provided to the public, and how these messages may be perceived by different groups, avoiding an overload of information where possible.

In addition, it should be noted that participants further expressed a lack of trust in government officials over time, due to the ever-changing guidance that led them to believe the government was disorganised in tackling the pandemic. In contrast, results from a recent survey showed that trust in government was of little importance in predicting compliance [36]. However, it should be noted that this survey used pre-designed, closed-questions, and was collected internationally. Therefore, it is unclear whether participants from different countries expressed the same governmental concerns, or whether their views changed over time. In comparison, the current study employed in-depth, qualitative methods at two separate timepoints during the pandemic, allowing for a rich exploration of influencing factors that may have changed over time. Moreover, loss of trust in the government impacting compliance with pandemic restrictions has been reported elsewhere [40, 41]. However, the results from our study highlight that this finding also applied to individuals classed as highly vulnerable to the virus, including those from deprived areas. Where individuals did not trust the source of guidance, their risk perception around their vulnerable status decreased and so, they were less likely to comply.

A further finding from our research was the varied perception of risk to self versus the risk perceived to others. Specifically, age was a frequent demographic factor compared between groups. Despite national guidance declaring older people to be highly vulnerable to the virus, previous evidence reported that elderly people felt significantly less likely to become infected with the virus than younger people [9]. The authors failed to suggest reasons for this finding. However, our current research offers an explanation, as elders did not appear to view older age to be a risk factor if they considered themselves to be in good health. Elders as a population, are more likely to experience health conditions that increase their risk of becoming infected, but for those without health conditions, their view on generalised guidance was less clear. Therefore, even where participants were classed as vulnerable to the adverse effects of the virus, their perception of risk to self was individually constructed through their own opinion of their health, which offers insight into how government guidance was perceived, and subsequently complied with, by this group.

Through the comparisons made between individuals, a shame and blame culture around compliance with restrictions emerged in participant accounts, such as the observation of others failing to comply with guidance on the use of face coverings and social distancing. An Australian survey reported that compliance rates dropped the longer the public experienced lockdown, although explanation for this finding was not collected [42]. However, our study found contrasting experiences of non-compliance in the early days of the pandemic, whereby participants strived to fit in with the actions of their peers, irrespective of risk perception. A study among younger adults in Switzerland with a history of delinquent behaviours, or association with delinquent peers, reported they were less likely to comply with pandemic restrictions. However, our study highlights that this finding extends to older groups, including those who simply wanted to avoid the judgement of others. Furthermore, the heightened use of social media during the pandemic to stigmatise the actions of others [43] further defends the argument that scrutiny of peoples’ actions during the pandemic was widespread, and so a desire to adhere to peer opinion may have superseded one’s own desire to comply with risk prevention measures.

However, as COVID-19 rates and subsequent perceived risk increased over time, individuals began to question the actions of others, and called for greater levels of policing. Sociological evidence has found social shaming to provide people with a sense of community in times where this is needed [44], which when considered in the context of social isolation during the pandemic, may have provided participants with a sense of reassurance and bonding with others. Thus, it is apparent that people did not remain either compliant or non-compliant during the pandemic [45], and instead, considered the changing social and personal motivations during the course of the pandemic to influence compliance. This finding further draws on the theory of social interactionism, which states that individuals consider behaviour and social roles to help understand and react to their environment [24]. Our research identified this phenomenon when participants considered the behaviours and beliefs of others around them (such as, family, colleagues, and authority) when deciding whether to comply with pandemic guidance, or conform to social norms. Further evidence showed that countries with tight cultures and stricter punishments for deviance, had fewer cases and deaths from COVID-19 compared with loose cultures, which have weaker norms and are more permissive [46]. In our study, participants reported a lack of consequence from the UK government when breaches in guidance were observed, and highlights the impact cultural context has on compliance. Understanding how guidance is perceived and followed, and the social or physical influences that hinder compliance, provides governing bodies with deeper insight into the feasibility of restrictions at that time.

Overall, this research has highlighted that participants created their own understanding of COVID-19 risk perception through personal experience and comparison with others around them, irrespective of vulnerability status. This is explained well within the theory of symbolic interactionism, where participants considered the views of the local community and their immediate contacts, in addition to the expectations of local authority and their representatives. Depending on how individuals and others of influence behaved and expressed their views, government guidance was therefore, not always followed as intended and, at times, even rejected due to lack of trust. Therefore, governments must consider the format in which future pandemic guidance is conveyed, and consider individuals’ experiences that may lead to non-compliance, and the power of social interactionism. We suggest the following recommendations from our findings:

1. Government pandemic guidance should aim to be delivered clearly and consistently, with rationale provided to enhance compliance across groups. Where individuals do not understand the guidance, or trust the sources, they will cease to follow it.
2. Classing individuals into different risk categories, with different levels of restrictions, may not be accepted by the individuals, leading to non-compliance and judgement of others. Wider consideration should be given to individual factors that make up one’s ability and choice to comply with pandemic guidance.
3. Considering the varied perception of risk and compliance between individuals, future policies on recovery from the pandemic must also consider personal experiences, and take an individualised approach.

## Limitations

The findings from this research are limited to views of participants in one region within North West England. None of our sample self-described as being from an ethnic minority, but the proportions of residents described as such in the census is relatively low within the areas sampled for our study. A separate study exploring perception of risk in Muslim communities was conducted as part of an overarching research study, and so these experiences have been documented elsewhere [8, 47].

## Conclusions

Our research identified that participants developed their own understanding of COVID-19 risk perception through personal experience and comparison with others around them, irrespective of government-classed vulnerability status. These findings highlight the complexity of one’s perception of risk, which is multifaceted and can alter over time as a result of media sources, government trust and socially (through the actions and views of others). As a result, COVID-19 guidance was not always complied with as intended by the government, and at times, was even rejected by the individual due to lack of trust. The format in which future pandemic guidance is conveyed must be carefully considered, as unclear and inconsistent guidance precludes compliance. Furthermore, individuals’ personal circumstances and experiences must be considered, as risk perception appears to be individually constructed and may change over time. If individuals feel their personal circumstances, caring responsibilities, or the environment in which they live renders compliance impossible, or observe a lack of policing where others non-comply, mistrust in government leadership and a rejection of the guidance may follow.

## Supplementary Information

**Additional file 1:** **Supplementary Material 1.** Timeline of pandemic restrictions in the UK (1, 2).

**Additional file 2.** COREQ (COnsolidated criteria for REporting Qualitative research) Checklist.

**Additional file 3:** **Appendix 1.** Topics guides.

**Additional file 4.**

## Data Availability

The datasets generated and/or analysed during the current study are not publicly available due to ethical requirements to maintain anonymity, but are available from the corresponding author on reasonable request.

## References

[1] Sohrabi C, Alsafi Z, O'neill N, Khan M, Kerwan A, Al-Jabir A (2020). World Health Organization declares global emergency: A review of the 2019 novel coronavirus (COVID-19). Int J Surg.

[2] Hodgson K, Peytrignet S (2021). Who was advised to shield from COVID-19?.

[3] Gov UK (2020). Staying at home and away from others (social distancing) London: Cabinet Office.

[4] Darker C, Gellman MD, Turner JR (2013). Risk Perception. Encyclopedia of Behavioral Medicine.

[5] Rohrmann B. Risk perception, risk attitude, risk communication, risk management: A conceptual appraisal. 15th Internaional Emergency Management Society (TIEMS) Annual Conference; Melbourne: 2008.

[6] Cori L, Bianchi F, Cadum E, Anthonj C (2020). Risk Perception and COVID-19. Int J Environ Res Public Health.

[7] Kirby T (2020). Evidence mounts on the disproportionate effect of COVID-19 on ethnic minorities. Lancet Respir Med.

[8] Hassan SM, Ring A, Tahir N, Gabbay M (2021). How do Muslim community members perceive Covid-19 risk reduction recommendations - a UK qualitative study?. BMC Public Health.

[9] Gerhold L (2020). COVID-19: risk perception and coping strategies.

[10] Wise T, Zbozinek TD, Michelini G, Hagan CC, Mobbs D (2020). Changes in risk perception and self-reported protective behaviour during the first week of the COVID-19 pandemic in the United States. R Soc Open Sci.

[11] Cheng Z, Mendolia S, Paloyo AR, Savage DA, Tani M (2021). Working parents, financial insecurity, and childcare: mental health in the time of COVID-19 in the UK. Rev Econ Household.

[12] Emerson E, Stancliffe R, Hatton C, Llewellyn G, King T, Totsika V (2021). The impact of disability on employment and financial security following the outbreak of the 2020 COVID-19 pandemic in the UK. J Public Health.

[13] Power M, Doherty B, Pybus K, Pickett K. How COVID-19 has exposed inequalities in the UK food system: The case of UK food and poverty. Emerald Open Research. 2020;2(11):1-26.

[14] Patel J, Nielsen F, Badiani A, Assi S, Unadkat V, Patel B (2020). Poverty, inequality and COVID-19: the forgotten vulnerable. Public Health.

[15] Varma P, Junge M, Meaklim H, Jackson ML (2021). Younger people are more vulnerable to stress, anxiety and depression during COVID-19 pandemic: A global cross-sectional survey. Prog Neuropsychopharmacol Biol Psychiatry.

[16] Tuthill EL, Maltby AE, DiClemente K, Pellowski JA (2020). Longitudinal Qualitative Methods in Health Behavior and Nursing Research: Assumptions, Design, Analysis and Lessons Learned. Int J Qual Methods.

[17] Richard VM, Lahman MKE (2015). Photo-elicitation: reflexivity on method, analysis, and graphic portraits. Int J Res Method Educ.

[18] Corcoran R, Ring A, Hassan S, Abba K, Downing J, Goodall M (2022). Characteristics of mental health stability during COVID-19: An online survey with people residing in a city region of the North West of England. PloS ONE.

[19] National Health Service (2022). Who is at high risk from coronavirus (COVID-19): National Health Service.

[20] National Health Service (2022). Deprivation London: NHS 75 England.

[21] Tong A, Sainsbury P, Craig J (2007). Consolidated criteria for reporting qualitative research (COREQ): a 32-item checklist for interviews and focus groups. Int J Qual Health Care.

[22] Glaw X, Inder K, Kable A, Hazelton M (2017). Visual methodologies in qualitative research: Autophotography and photo elicitation applied to mental health research. Int J Qual Methods.

[23] Hill R, Betts LR, Gardner SE (2015). Older adults’ experiences and perceptions of digital technology: (Dis)empowerment, wellbeing, and inclusion. Comput Hum Behav.

[24] Carter MJ, Fuller C (2016). Symbols, meaning, and action: The past, present, and future of symbolic interactionism. Curr Sociol.

[25] Braun V, Clarke V (2006). Using thematic analysis in psychology. Qual Res Psychol.

[26] Clarke V, Braun V. Thematic analysis: a practical guide. London: Sage Publications; 2021.

[27] Braun V, Clarke V (2019). Reflecting on reflexive thematic analysis. Qual Res Sport Exerc Health.

[28] QSR International pty Ltd. Nvivo 12 Software. Melbourne. 2012.

[29] Dodgson JE (2019). Reflexivity in Qualitative Research. J Hum Lact.

[30] Berger R (2015). Now I see it, now I don’t: Researcher’s position and reflexivity in qualitative research. Qual Res.

[31] Staniszewska S, Brett J, Simera I, Seers K, Mockford C, Goodlad S (2017). GRIPP2 reporting checklists: tools to improve reporting of patient and public involvement in research. RIE.

[32] National Statistics (2019). English Indices of Deprivation 2019 London: Ministry of Housing, Communiities and Local Government.

[33] Whitehead M (2014). Due North.

[34] UK Health Security Agency (2021). COVID-19: guidance for people whose immune system means they are at higher risk London: Deartment of Health and Social Care.

[35] Williams SN, Armitage CJ, Tampe T, Dienes KA (2021). Public perceptions of non-adherence to pandemic protection measures by self and others: A study of COVID-19 in the United Kingdom. PLoS ONE.

[36] Clark C, Davila A, Regis M, Kraus S (2020). Predictors of COVID-19 voluntary compliance behaviors: An international investigation. Global transitions.

[37] Motta Zanin G, Gentile E, Parisi A, Spasiano D (2020). A Preliminary Evaluation of the Public Risk Perception Related to the COVID-19 Health Emergency in Italy. Int J Environ Res Public Health.

[38] Montiel CJ, Uyheng J, Dela PE (2021). The Language of Pandemic Leaderships: Mapping Political Rhetoric During the COVID-19 Outbreak. Polit Psychol.

[39] Moss SM, Sandbakken EM (2021). “Everybody Needs to Do Their Part, So We Can Get This Under Control.” Reactions to the Norwegian Government Meta-Narratives on COVID-19 Measures. Polit Psychol.

[40] Lalot F, Heering MS, Rullo M, Travaglino GA, Abrams D (2020). The dangers of distrustful complacency: Low concern and low political trust combine to undermine compliance with governmental restrictions in the emerging Covid-19 pandemic. Group Process Intergroup Relat.

[41] Prati G, Pietrantoni L, Zani B (2011). Compliance with recommendations for pandemic influenza H1N1 2009: the role of trust and personal beliefs. Health Educ Res.

[42] Murphy K, Williamson H, Sargeant E, McCarthy M (2020). Why people comply with COVID-19 social distancing restrictions: Self-interest or duty?. Aust N Z J Criminol.

[43] Travaglino GA, Moon C (2021). Compliance and self-reporting during the COVID-19 pandemic: a cross-cultural study of trust and self-conscious emotions in the United States, Italy, and South Korea. Front Psychol.

[44] Scheff TJ (2000). Shame and the social bond: A sociological theory. Sociol Theory.

[45] McCarthy M, Murphy K, Sargeant E, Williamson H (2021). Policing COVID-19 physical distancing measures: managing defiance and fostering compliance among individuals least likely to comply. Polic Soc.

[46] Gelfand MJ, Jackson JC, Pan X, Nau D, Pieper D, Denison E (2021). The relationship between cultural tightness–looseness and COVID-19 cases and deaths: a global analysis. The Lancet Planetary Health.

[47] Hassan SM, Ring A, Tahir N, Gabbay M (2021). The impact of COVID-19 social distancing and isolation recommendations for Muslim communities in North West England. BMC Public Health.

[48] Atchison C, Bowman LR, Vrinten C, Redd R, Pristerà P, Eaton J, Ward H (2021). Early perceptions and behavioural responses during the COVID-19 pandemic: a cross-sectional survey of UK adults. BMJ Open.

[49] Barrios JM, Hochberg Y. Risk perception through the lens of politics in the time of the COVID-19 pandemic. Cambridge: National Bureau of Economic Research; 2020.

[50] de Bruin W (2021). Age differences in COVID-19 risk perceptions and mental health: Evidence from a national US survey conducted in March 2020. J Gerontol Series B.

[51] de Bruin WB, Bennett D (2020). Relationships between initial COVID-19 risk perceptions and protective health behaviors: a national survey. Am J Prev Med.

[52] Olmos-Vega FM, Stalmeijer RE, Varpio L, Kahlke R. A practical guide to reflexivity in qualitative research: AMEE Guide No. 149. Medical teacher. 2023;45(3):241-51. 10.1080/0142159X.2022.2057287 35389310

[53] Rosi A, Van Vugt FT, Lecce S, Ceccato I, Vallarino M, Rapisarda F, Vecchi T, Cavallini E (2021). Risk perception in a real-world situation (COVID-19): how it changes from 18 to 87 years old. Front Psychol.

[54] Sherman SM, Smith LE, Sim J, Amlôt R, Cutts M, Dasch H, Rubin GJ, Sevdalis N (2021). COVID-19 vaccination intention in the UK: results from the COVID-19 vaccination acceptability study (CoVAccS), a nationally representative cross-sectional survey. Hum Vaccin Immunother.

[55] Schneider CR, Dryhurst S, Kerr J, Freeman AL, Recchia G, Spiegelhalter D, van der Linden S (2021). COVID-19 risk perception: a longitudinal analysis of its predictors and associations with health protective behaviours in the United Kingdom. J Risk Res.

[56] Tsoy D, Tirasawasdichai T, Kurpayanidi KI (2021). Role of social media in shaping public risk perception during COVID-19 pandemic: A theoretical review. Int J Manag Sci Bus Adm.

