# Supplementary material for: The perception of risk in contracting and spreading COVID-19 amongst individuals, households and vulnerable groups in England: a longitudinal qualitative study
Source: BMC Public Health. 2023 Apr 5;23:653. doi: 10.1186/s12889-023-15439-8 (PMC10074336; doi:10.1186/s12889-023-15439-8)
Supplement: Supplementary file 1 — Additional file 1: Supplementary Material 1. Timeline of pandemic restrictions in the UK (1, 2). [file 12889_2023_15439_MOESM1_ESM.docx]

***Supplementary Material 1: Timeline of pandemic restrictions in the UK (1, 2)***

1. ***Prior to the data collection period (March to July)***

National lockdown was announced on March 23^rd^ with people told to stay at home except for limited reasons including going to work if they could not work at home, essential shopping, caring responsibilities, medical needs. Outdoor exercise (alone or with household members) with allowed once per day, and public gatherings banned. Schools were closed to most children, ‘non-essential’ shops and services were closed, and many jobs and services were adapted to enable home working. People who were ‘extremely clinically vulnerable’ to COVID were recommended to ‘shield’ and not leave their home at all, even for exercise. This gradually relaxed over the summer. In May, limits on the amount of outdoor exercise were relaxed and people could meet up with one other person outside. In June, restrictions were further eased with people allowed to travel further, spend more time outdoors, and meet with others in groups of up to six outdoors. ‘Support bubbles’ were introduced in June to allow people in single-adult households to meet with people in other single adult households, although people who were shielding were advised not to form support bubbles. Schools re-opened to some year-groups, and ‘non-essential’ shops and places of worship also re-opened. In July two households of any size were allowed to meet in any location. Including indoors. More amenities re-opened subject to safety rules, including playgrounds, museums, pubs, restaurants, hairdressers, sports facilities and wedding venues. People who were shielding were advised that they could form a support bubble and meet up with others in groups of six outdoors. On July 4^th^, the first local lockdown was announced in Leicester as restrictions were eased in other parts of England including the re-opening of pubs, restaurants and hairdressers.

In March, COVID testing was reserved for hospital patients with respiratory symptoms. During April, symptomatic testing was expanded to social care staff and clients, then healthcare staff and their household members, then anybody who had to leave the house to work or was over the age of 65.

Initially, recommendations for the public advised against wearing face coverings. In May, guidance was introduced recommending people to wear face coverings in crowded indoor settings. In June face-coverings were made compulsory in public transport and hospitals and in July they were made compulsory in shops, except for children and people who could not wear face covering for medical reasons.

Test and Trace was introduced at the end of May, with the legal responsibility of people who tested positive and their recent contacts to self-isolate for 14 days.

1. ***During the data collection period***

During the period of data collection, the “Eat out to help out scheme” was launched (3^rd^ August); lockdown restrictions were eased allowing people to sit indoors (14^th^ August); rule of six people allowed to gather indoors/outdoors was banned in England (14^th^ September); 10pm curfew announced in England (22^nd^ September); a new three-tiered restriction system announced (14^th^ October); and a second lockdown announced (31^st^ October).

1. Institute for Government. Timeline of UK government coronavirus lockdowns and restrictions. London: Institute for Government; 2022.

2. Brown J, Kirk-Wade E. Coronavirus: A history of 'Lockdown laws' in England. London: House of Commons Library; 2021.
