# Supplementary material for: The perception of risk in contracting and spreading COVID-19 amongst individuals, households and vulnerable groups in England: a longitudinal qualitative study
Source: BMC Public Health. 2023 Apr 5;23:653. doi: 10.1186/s12889-023-15439-8 (PMC10074336; doi:10.1186/s12889-023-15439-8)
Supplement: Supplementary file 3 — Additional file 3: Appendix 1. Topics guides. [file 12889_2023_15439_MOESM3_ESM.docx]

Appendix 1: Topics guides

1. **Topic Guide: Baseline interviews**

**Understanding of COVID-19/coronavirus**

So I’ll start first by asking just a few questions about your understanding of the coronavirus, there are no right or wrong answers, we are only interested in your views.

- *So first, can I ask you* how you would describe what COVID-19/Coronavirus is?
- *How do you think the virus is passed from one person to another?*
- Where do you get information about COVID-19/ coronavirus from?

**Risk and impact**

*Do you think you are at risk of contracting the virus? Why?*

Prompt: Worries/fears (if any) in case they contract the virus.

*Thinking about other members of your household, do you think the risks are different for them? Why?*

Prompt: Worries/fears (if any) in case household members contract the virus.

*Thinking about family and friends not living within your household, do you think the risks are different for them? Why?*

Prompt: Worries/fears (if any) in case family members/friends not living in house contract virus

*How, if at all, have your views about risk changed from when lockdown started in March?*

**Self-isolation**

*Did you have had to self-isolate because of symptoms that you or someone in your household have had?*

*If yes:* What has been your experience of trying to self-isolate?

Prompts*:*

- Particular challenges and their impact on ability to self-isolate
- Anything that helped with self-isolation

**Social distancing**

*The government have advised that social distancing can reduce the risk of COVID19.What do you think about this advice, thinking perhaps first about advice at the start of lockdown?*

Prompts:

- Impact of advice on life
- Worries about restrictions in place at that time
- Anything that made following advice difficult
- Anything that made social distancing easier
- Anything that participant or household members have done to reduce risk of getting the virus.
- Particular issues around shielding

What do you think about the more recent changes to social distancing advice?

Prompts:

- Impact of advice (positive and/or negative)
- Changing impact
- Views about use of masks/face covering

**Information and advice**

*Where have you been getting information about COVID-19 and the pandemic?*

Prompt: Sources of information trust/distrust - Why?

*Can I ask you now, what you think in general about the information that has been coming out from UK government?*

*How do you think this information could have been improved?*

Prompts:

- To inform people better about how they could reduce risks to themselves and others
- To help them understand what the government was asking of them and what the rules were

*Is there anything that I haven’t asked about that you would like to add?*

1. **Follow-up interview topic guide**

We would like to understand your experience of managing risk related to COVID-19 over the last four weeks since we last spoke with you, and the impact of the government's social distancing recommendations on the way you and others in your household live your lives.

How have you been since we last spoke? And those close to you?

Has anything changed for you?

What has changed?

What effect has the changed had on your life?

Has anything changed for other members of your household or family?

What has changed?

What effect has the changed had on your life?

Have you noticed any other changes around you, for example in your local community?

What has changed?

What do you think of these changes?

Are there any other changes that you would like to see happen soon?

What changes? Why?

What do you think could be done to help people recover once the crisis has passed?

Is there anything else you would like to tell us about how the COVID-19 pandemic and the social distancing directives have affected your life, or other people’s lives?

1. **Photo elicitation topic guide, if applicable, for follow up interview**

How have you been since we last talked? And those close to you?

How has the process of taking photographs over the last 4 weeks been for you? Did you discuss the photographs with other people in your household or community?

**Exploring experiences of risk and risk reduction measures**

***What thoughts and ideas have your photographs brought out for you about the COVID-19 pandemic and risk associated with it?***

Can you talk me through the photographs you’ve taken that you think best illustrate your experience of managing risk related to COVID-19 and the effect of the current social distancing recommendations on the way that you and others in household live your lives. We will probably have time to talk about three to five photographs. Start with the photograph that means most to you**.**

*For each photograph, ask:*

Please tell me about this photo (context, why, what, who)

Why did you choose this photograph?

What does it show or represent?

Are the changes you describe the same as when you took the photo or has anything change since you took the photograph?

Who is most affected by the changes it shows?

- If something negative - is there anything you think could make it better?
- If something positive - would you like to see that continue after the current crisis has passed?

Is there anything else that you would like to tell us about your experiences of this covid19 pandemic or the effects of the social distancing policies for you and your household or family?
