# Supplementary material for: The perception of risk in contracting and spreading COVID-19 amongst individuals, households and vulnerable groups in England: a longitudinal qualitative study
Source: BMC Public Health. 2023 Apr 5;23:653. doi: 10.1186/s12889-023-15439-8 (PMC10074336; doi:10.1186/s12889-023-15439-8)
Supplement: Supplementary file 4 — Additional file 4. [file 12889_2023_15439_MOESM4_ESM.docx]

Supplementary material 3

**Bibliography**

- Atchison C, Bowman LR, Vrinten C, Redd R, Pristerà P, Eaton J, Ward H. Early perceptions and behavioural responses during the COVID-19 pandemic: a cross-sectional survey of UK adults. BMJ open. 2021.11(1):e043577.
- Barrios JM, Hochberg Y. Risk perception through the lens of politics in the time of the COVID-19 pandemic. National Bureau of Economic Research; 2020.
- de Bruin W. Age differences in COVID-19 risk perceptions and mental health: Evidence from a national US survey conducted in March 2020. The Journals of Gerontology: Series B. 2021 Feb;76(2):e24-9.
- de Bruin WB, Bennett D. Relationships between initial COVID-19 risk perceptions and protective health behaviors: a national survey. American Journal of Preventive Medicine. 2020 Aug 1;59(2):157-67.
- Olmos-Vega FM, Stalmeijer RE, Varpio L, Kahlke R. A practical guide to reflexivity in qualitative research: AMEE Guide No. 149. Medical teacher. 2022
- Rosi A, Van Vugt FT, Lecce S, Ceccato I, Vallarino M, Rapisarda F, Vecchi T, Cavallini E. Risk perception in a real-world situation (COVID-19): how it changes from 18 to 87 years old. Frontiers in psychology. 2021 2;12: 646558.
- Sherman SM, Smith LE, Sim J, Amlôt R, Cutts M, Dasch H, Rubin GJ, Sevdalis N. COVID-19 vaccination intention in the UK: results from the COVID-19 vaccination acceptability study (CoVAccS), a nationally representative cross-sectional survey. Human vaccines & immunotherapeutics. 2021 3;17(6):1612-21.
- Schneider CR, Dryhurst S, Kerr J, Freeman AL, Recchia G, Spiegelhalter D, van der Linden S. COVID-19 risk perception: a longitudinal analysis of its predictors and associations with health protective behaviours in the United Kingdom. Journal of Risk Research. 2021 22;24(3-4):294-313.
- Tsoy D, Tirasawasdichai T, Kurpayanidi KI. Role of social media in shaping public risk perception during COVID-19 pandemic: A theoretical review. International Journal of Management Science and Business Administration. 2021 7;7(2):35-41.
